# Phenotypic and genomic characterization of vB_SauP-INT105, an exopolysaccharide depolymerase-encoding lytic short-tailed phage with antibiofilm activity against *Staphylococcus aureus*

**DOI:** 10.3389/fmicb.2026.1856705

**Published:** 2026-06-17

**Authors:** Joaquín Rodríguez-Pinilla, Alfredo García, Felipe Molina, Ana Magdalena, Lucía Fernández, Pilar García, Rafael Tabla

**Affiliations:** 1 Centro de Investigaciones Científicas y Tecnológicas de Extremadura (CICYTEX), Badajoz, Spain; 2 Departamento de Bioquímica y Biología Molecular y Genética, Universidad de Extremadura, Badajoz, Spain; 3 Instituto de Productos Lácteos de Asturias (IPLA-CSIC), Oviedo, Spain

**Keywords:** biocontrol, biofilm, exopolysaccharide depolymerase, lytic bacteriophage, Rosenblumvirus, *Staphylococcus aureus*

## Abstract

**Introduction:**

*Staphylococcus aureus* is a major opportunistic pathogen associated with dairy production systems, where it contributes to intramammary infections and may enter the food chain through contaminated milk. The increasing prevalence of antimicrobial-resistant strains underscores the need for alternative biocontrol strategies. Here, we report the isolation and comprehensive characterization of a novel lytic bacteriophage, vB_SauP-INT105, obtained from wastewater in Extremadura, Spain.

**Methods and results:**

Plaque morphology revealed a distinctive three-zone halo pattern suggestive of phage-encoded exopolysaccharide depolymerase activity, confirmed by bioinformatic prediction of two candidate depolymerase-encoding ORFs. Electron microscopy showed a bacteriophage with an icosahedral head and a short non-contractile tail. The phage exhibited lytic activity against 86.5% of 37 dairy-associated *S. aureus* isolates tested. One-step growth analysis revealed a latency period of ~40 minutes and a burst size of ~23 PFU/cell. The phage showed robust stability across a wide range of temperatures and pH values relevant to dairy-processing conditions, retaining viability over 20 months at 4 °C. Genome sequencing revealed a 17.45 kb dsDNA genome encoding 20 ORFs, with no detectable genes associated with virulence, lysogeny, or antibiotic resistance. Phylogenetic analysis placed INT105 as a novel species within the *Rosenblumvirus* genus. Antibiofilm assays demonstrated significant reductions in viable cell counts in two *S. aureus* strains with contrasting biofilm phenotypes.

**Conclusion:**

These results establish INT105 as a genomically safe, environmentally robust and functionally effective phage with strong potential for biocontrol applications in dairy-production environments and broader food-safety and clinical contexts.

## 1 Introduction

*Staphylococcus aureus* is a major opportunistic pathogen of clinical and veterinary relevance, responsible for infections ranging from cutaneous abscesses and mastitis to endocarditis and bacteremia (Peng et al., 2023). In small ruminants, staphylococci represent the primary etiological agents of intramammary infections, with *S. aureus* being the species most frequently isolated in clinical mastitis cases (Bergonier et al., 2003; Tibebu et al., 2025). Traditionally, the therapeutic management of these infections has relied on antibiotics. However, the ability of *S. aureus* to acquire multiple antimicrobial resistance mechanisms has made it an increasing challenge for public health (Atshan et al., 2023; Bowden et al., 2024). Moreover, *S. aureus* is a leading cause of foodborne disease linked to the consumption of contaminated milk and dairy products (Campos et al., 2022). Entry into the human food chain can occur at the farm through the milking of infected animals, or later during processing, via human handlers or contaminated surfaces in contact with milk (Oliveira et al., 2022; Kümmel et al., 2016). The strong biofilm-forming capacity of *S. aureus*, mediated by an extracellular matrix composed of polysaccharides, proteins, and DNA, further enhances its resistance to antimicrobial agents and facilitates its persistence on biological and inert surfaces (Peng et al., 2023; Zhao et al., 2023).

In a scenario marked by limited progress in developing new antibiotics and the rise of resistant strains, whose persistence is further exacerbated by their biofilm-forming capacity, there is growing interest in exploring alternative therapeutic and biocontrol strategies. Among these, bacteriophages represent a promising approach due to their ability to lyse bacteria with high specificity and their potential to disrupt biofilms (Fernández et al., 2019; Atshan et al., 2023). Phages suitable for therapeutic or biocontrol use must be strictly lytic and ideally display a broad host range (Picozzi et al., 2021). Additional desirable traits include a high adsorption rate (Abedon, 2023), short latent period, and large burst size (Meneses et al., 2023). Environmental stability ensures both phage preservation and effective application under the temperature and pH conditions characteristic of the technological processes or matrices in which it is intended for use (Jayamanne and Foddai, 2025). Furthermore, candidate phages must lack genes encoding virulence factors or antibiotic resistance and exhibit a low potential to transfer such genes via transduction (Cook and Hynes, 2025). The production of depolymerases capable of degrading exopolysaccharides constitutes an additional desirable trait, as these enzymes contribute to biofilm disruption and enhance the penetration and diffusion of antimicrobial agents, increasing their effectiveness in both clinical and food-related environments (Oliveira et al., 2022; Guo et al., 2023).

Phages infecting *S. aureus* have historically been described as tailed viruses and were previously classified into the families *Podoviridae*, *Siphoviridae*, and *Myoviridae*, all of which were contained within the former order *Caudovirales* (Azam and Tanji, 2019; Deghorain and Van Melderen, 2012). However, recent ICTV taxonomic updates have abolished these classical morphological families. This reclassification replaced the order *Caudovirales* with the class *Caudoviricetes*, a phylogenetically defined lineage encompassing all tailed viruses with an icosahedral capsid and double-stranded DNA, and introduced several new families (Turner et al., 2023). These updates illustrate the limitations of morphology-based classification in capturing the phylogenetic diversity of *S. aureus* phages and other bacteriophages.

Several studies have explored the use of bacteriophages as biocontrol agents against *S. aureus* in dairy matrices (Bueno et al., 2012; El Haddad et al., 2016; Kwak et al., 2023; Magdalena et al., 2025). Nevertheless, despite the growing interest in phage-based biocontrol in dairy systems, the isolation and characterization of lytic phages specifically active against *S. aureus* strains from traditional dairy environments remain comparatively underexplored, highlighting the need for targeted efforts to identify phage candidates suited to these particular ecological and technological contexts. In this study, we report the isolation and comprehensive characterization of the lytic phage vB_SauP-INT105, a member of the *Rountreeviridae* family within the class *Caudoviricetes*. Given the taxonomic and ecological diversity of this class, many of its members remain poorly characterized regarding their suitability for biocontrol applications. vB_SauP-INT105 exhibited activity against multiple *S. aureus* isolates from the tested collection, favorable lysis kinetics, notable environmental stability, and the ability to kill bacteria within biofilms, making it a promising candidate for controlling *S. aureus* in both clinical and industrial contexts.

## 2 Materials and methods

### 2.1 Bacterial strains and culture conditions

This study utilized 37 *S. aureus* isolates (Supplementary Table S1) obtained from dairy goat farms in the Extremadura region of Spain in 2022 and preserved at −80 °C in the culture collection of the Center for Scientific and Technological Research of Extremadura (CICYTEX). The isolates were originally recovered on Baird–Parker Egg Yolk Tellurite Agar (Oxoid, Basingstoke, United Kingdom) based on characteristic colony morphology and presumptively identified as *S. aureus* by the coagulase test. For this study, isolates were routinely cultured in tryptic soy broth (TSB, Oxoid, Basingstoke, United Kingdom) or tryptic soy agar (TSA, Oxoid, Basingstoke, United Kingdom) at 37 °C.

### 2.2 Phage isolation and phage stock preparation

A total of 48 water samples (100 mL each) were collected between July 2022 and May 2023 for phage isolation. These samples were obtained from the influent of various wastewater treatment plants located in the province of Badajoz (Extremadura, Spain) and provided by a water analysis company. Due to the confidentiality and traceability protocols of the provider, samples were identified using internal codes. Upon arrival, the samples were centrifuged at 12,000 × *g* for 15 min and then filtered through a 0.22 μm cellulose acetate filter (VWR, Radnor, United States). Phage isolation was performed as previously described by Khan and Joshi (2022) with minor modifications. Briefly, phage enrichment and amplification were performed using *S. aureus* strains isolated from goat milk as the bacterial host. Ten mL of the filtered wastewater sample was mixed with an equal volume of 2 × TSB. This mixture was inoculated with 1 mL of an overnight culture of the *S. aureus* strain and incubated at 30 °C for 24 h with agitation, followed by storage at 4 °C for 24 h.

Phage quantification was performed by the double-layer agar (DLA) method (Kropinski et al., 2009) on TSA plates. The *S. aureus* strain previously used for phage amplification served as the indicator host. Phage plaques, formed after 24 h at 37 °C, were purified by successive pick-ups until consistent plaque size and morphology were achieved. To obtain high-titer phage stocks, 4 mL of SM buffer (Tris–HCl 20 mM pH 7.5; NaCl 100 mM; MgSO_4_ 10 mM; Ca(NO_3_)_2_ 10 mM) was added to semi-confluent growth plates. After 6 h of shaking at room temperature, the phage suspension was collected and centrifuged at 12,000 × *g* for 15 min. The supernatant was filtered through a 0.22 μm cellulose acetate filter. Bacteriophage stocks were titrated, aliquoted, and stored at 4 °C with 1% trichloromethane. Once stable phage stocks were established, plaque morphology was further evaluated by DLA method after 24 and 48 h of incubation at both 30 °C and 37 °C.

### 2.3 Phage morphology

The bacteriophage morphology and size were determined by electron microscopy. For negative staining, 15 μL of each sample were adsorbed to carbon-coated collodion 400 mesh copper grids (Gilder Grids Ltd., Grantham, United Kingdom) for 2 min, washed in two drops of water and stained with 2% aqueous uranyl acetate (Electron Microscopy Sciences, Hatfield, United States) for 1 min. Grids were visualized in a JEOL JEM 1400 Flash electron microscope (JEOL, Tokyo, Japan) operating at 100 kV. Micrographs were taken with a Gatan OneView digital camera (Gatan, California, United States) at various magnifications. Dimensions of phage particles (head diameter and tail length) were determined on micrographs at 50Kx or 80Kx magnification, with Gatan Micrograph Digital Software (Gatan Inc., Pleasanton, United States).

### 2.4 Host range

Host range was determined by cross-streaking (Molina et al., 2020). Briefly, a phage suspension (10^7^ PFU/mL) was applied in parallel lines on TSA plates, dried for 5 min and crossed perpendicularly with each *S. aureus* suspension (10^8^ CFU/mL). Plates were incubated overnight at 37 °C and examined. Zones of bacterial lysis were assessed with a scaling system, where 0 indicated no infection and 3 indicated a fully or nearly fully degraded bacterial lawn. Assays were performed in triplicate.

### 2.5 Phage adsorption assay

vB_SauP-INT105 phage was added to a culture of the sensitive strain Sa296 at a multiplicity of infection (MOI) of 0.01. This bacterial strain was randomly selected from a pool of isolates previously identified as highly susceptible to the phage based on initial host range assays. The mixture was incubated at room temperature without shaking. Aliquots were collected at 1, 3, 5, 10, 20, 40, and 60 min. Each aliquot was immediately transferred to a tube containing chloroform (1% v/v final concentration), mixed, centrifuged (14,000 × *g*, 30 s), and filtered through a 0.22 μm filter. The titer of the unabsorbed phages in the supernatant was subsequently measured using the DLA method.

### 2.6 One-step growth curve

The one-step growth curve was performed at an MOI of 0.01. A phage suspension (10^6^ PFU/mL) and the sensitive strain Sa296 (10^8^ CFU/mL) were incubated for 15 min at 37 °C to allow phage adsorption. To remove non-adsorbed phage particles, the suspension was centrifuged at 12,000 × *g* for 10 min at 4 °C. The resulting pellet was washed twice with ice-cold sterile TSB. After the second wash, the pellet was resuspended in 10 mL of pre-warmed TSB (37 °C). An initial aliquot was collected immediately, and the remaining suspension was incubated with shaking at 37 °C. Additional aliquots were taken every 10 min over a 90-min period. Each aliquot was centrifuged immediately (14,000 × *g*, 1 min). Decimal dilutions of the supernatant were prepared in SM buffer, and phage titers were determined by the DLA method.

### 2.7 pH and thermal stability

For pH stability analysis, the vB_SauP-INT105 phage suspension (10^9^ PFU/mL) was diluted 1:10 in TSB with pH values ranging from 4 to 9. Samples were titrated after 1 and 24 h of incubation at 37 °C. Similarly, to address thermal stability, the phage suspension was incubated at varying temperatures (−20, 4, 28, 37, 50, 68 and 80 °C) for 1 and 24 h in TSB (pH 7.3 ± 0.2). In addition, to evaluate prolonged storage stability, the phage preparation was kept at 4 °C under sterile conditions and titrated after 6 and 20 months. In all cases, phage titers were determined using the DLA method with Sa296 as the host strain. Experiments assessing the effects of pH and temperature were performed in triplicate.

### 2.8 Genome sequencing and bioinformatics analysis

Phage DNA was purified as follows. First, bacterial DNA and RNA were removed from 250 μL of a phage suspension with an enzymatic cocktail, containing Benzonase (125 U) (Sigma-Aldrich, St. Louis, United States), RNase Cocktail (RNase A 5 U; RNase T1 200U) (Thermo Fisher Scientific, Waltham, USA), DNase I (10 U) (Thermo Fisher Scientific, Waltham, USA), and (5 U) TurboDNAse (Thermo Fisher Scientific, Waltham, United States). After 2 h of incubation at 37 °C, the sample was treated with phenol:chloroform (3:1) and homogenized by vortexing. The aqueous layer containing phage DNA was collected after centrifugation at 10,000 × *g* for 2 min, and then 1 volume of chloroform was added to remove possible traces of phenol. Again, the upper layer was collected, and DNA was precipitated by adding 1:10 volume of sodium acetate 3 M (pH 5.8) and 2.5 volumes of 100% ethanol and incubating for 30 min at −80 °C. Samples were centrifuged for 15 min at 4 °C and the resulting pellet was then washed once with 70% ethanol and once with 100% ethanol. Following centrifugation for 2 min at 10,000 × *g*., the supernatant was removed and DNA samples were dried at room temperature and then resuspended in MilliQ water.

Genome sequencing with the prepared samples was performed by FISABIO (Valencia, Spain) with an Illumina NextSeq platform (Illumina, San Diego, CA, United States). FASTQC v. 0.11.3 (Andrews, 2010) was used to assess read quality. Read trimming was performed using Trim Galore v0.6.5dev (Krueger, 2023), and genome assembly was carried out with Unicycler v0.4.8 (Wick et al., 2017) on the Bacterial and Viral Bioinformatics Resource Center (BV-BRC) server (Olson et al., 2023). Pilon v1.23 was utilized for assembly polishing (Walker et al., 2014). Assembly quality assessment was performed with Bandage 0.8.1 (Wick et al., 2015) and QUAST v5.2.0 (Mikheenko et al., 2018). Subsequently, checkV was used to determine the degree of genome completeness (Nayfach et al., 2020), and a genome map was generated using PhageScope (Wang et al., 2024). The phage genome was annotated with the BV-BRC web tool, and a genome map was obtained using PhageScope. Similarity of vB_SauP-INT105 to other bacteriophages was assessed by generating a phylogenetic tree with VIPTree and its taxonomic distance to the 20 most similar phages according to BLASTn analysis (Camacho et al., 2009) was determined using VIRIDIC (Moraru et al., 2020). Identification of candidate proteins with depolymerase activity was performed using DePP^1^.

The phage genome has been deposited in the GenBank database under accession number PZ019817.

### 2.9 Biofilm elimination assay

The antibiofilm activity of phage vB_SauP-INT105 was analyzed as previously described by Gutiérrez et al. (2015a) with some modifications. Briefly, overnight cultures of *S. aureus* strains Sa296 and Sa105 were diluted 1:100 in fresh TSB medium supplemented with 0.25% glucose. Two mL aliquots from these cell suspensions were used to inoculate each well of a 12-microwell polystyrene plate (Thermo Scientific, Nunc, Madrid, Spain). Biofilms were then allowed to grow for 24 h in a static incubator at 37 °C. After biofilm development, the planktonic phase was removed and the samples were washed twice with PBS (137 mM NaCl, 2.7 mM KCl, 10 mM Na_2_HPO_4_, 2 mM KH_2_PO_4_; pH 7.4) prior to treatment with a phage suspension containing 10^8^ PFU/mL (treatment) or PBS alone (untreated control). Samples were then incubated for 24 h at 37 °C. Afterwards, the planktonic phase was removed again from both treated and untreated wells, and all biofilms were washed with PBS. The number of viable cells was determined by scraping the attached cells in 1 mL of PBS and plating serial dilutions from the resulting suspensions on 2% TSA plates.

In some cases, after the washing steps, the total biomass of treated and untreated biofilms was compared by crystal violet staining (Herrera et al., 2007). To do that, 1 mL of 0.1% crystal violet was placed into each well and allowed to dye the biofilms for 14 min at room temperature. After that, the excess dye was washed with distilled water. The remaining dye was solubilized with 2 mL of 33% acetic acid prior to quantification by measuring the absorbance at 595 nm with a microplate reader Tecan Infinite M Nano (Tecan Trading AG, Seestrasse, Switzerland).

Assays were carried out with three independent biological replicates. Data were analyzed with Student’s *t*-test, and differences were considered statistically significant at *p* < 0.05.

## 3 Results

### 3.1 Isolation and morphological characterization

**Figure 1:**
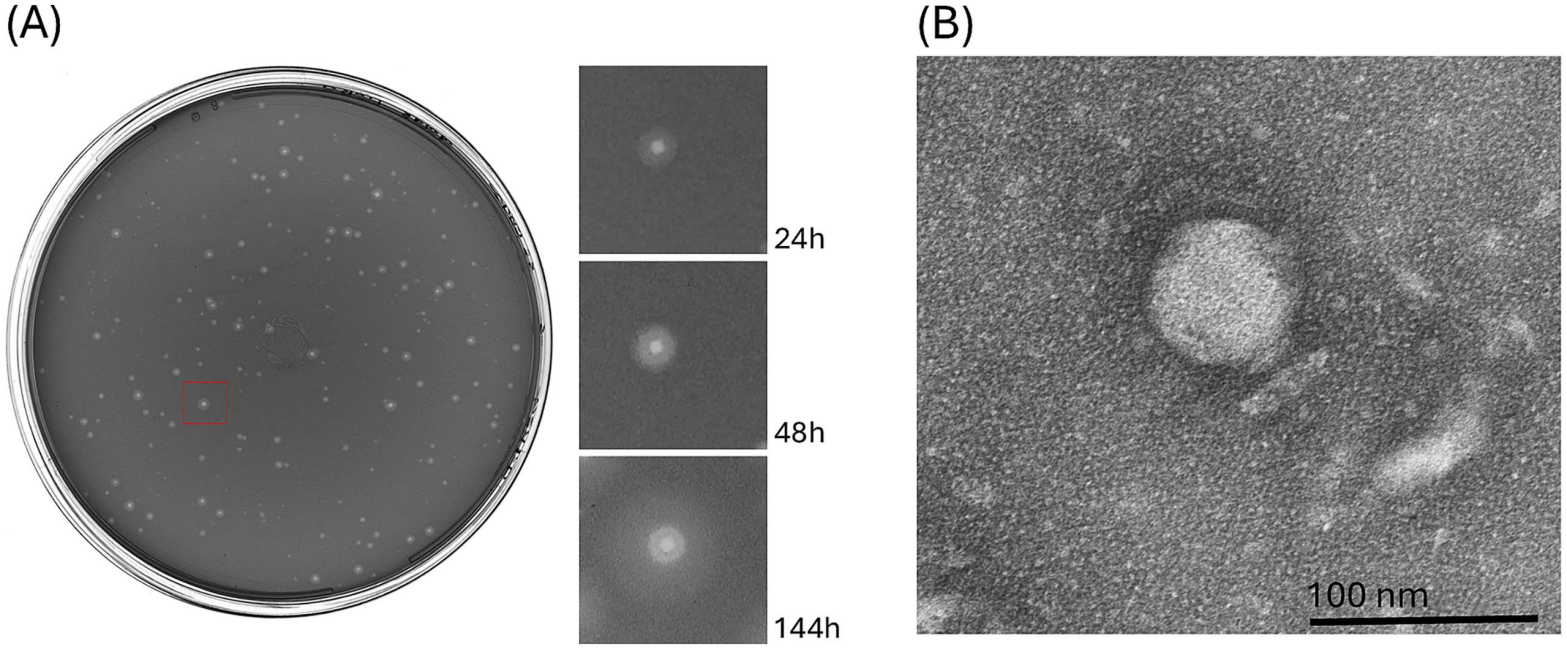
Plaque morphology and virion structure of bacteriophage vB_SauP-INT105 **(A)** Representative plaques produced by vB_SauP-INT105 on a Sa105 lawn, displaying well-defined zones of lysis. Insets illustrate plaque appearance at different incubation times (24 h, 48 h, and 144 h). **(B)** Transmission electron micrograph of negatively stained vB_SauP-INT105, revealing an icosahedral capsid and a short tail.

Phages were isolated from 48 samples collected from wastewater treatment plants in the Badajoz region (Extremadura, Spain). Plaque morphology was examined, looking for clear lytic plaques—indicative of bacterial lysis—surrounded by hazy peripheral haloes that may suggest depolymerase activity. While a total of seven phages were successfully isolated, six exhibited simple clear plaques without discernible haloes. Based on the presence of a distinct halo, one phage was selected for further characterization and designated as vB_SauP-INT105 (hereafter referred to as INT105). Phage plaques on *S. aureus* strain Sa105 developed translucent haloes starting at 24 h, reaching optimal definition and size after 48 h at 30 °C (Figure 1A). Detailed observation of halo dynamics revealed three zones of decreasing intensity from the center to the periphery. The two innermost zones stabilized at a diameter of approximately 2 mm after 48 h, remaining constant over time. In contrast, the outermost diffuse layer became discernible from 48 h onwards, gradually losing definition by 144 h of incubation. Electron microscopy revealed that INT105 had an icosahedral head with 46.89 ± 1.71 nm of width, a short non-contractile tail and a baseplate with 36.56 ± 6.11 (*n* = 5) of width (Figure 1B). This morphology is characteristic of tailed phages with short non-contractile tails, historically referred to as the podovirus morphotype (Ackermann, 2011).

### 3.2 Bacterial host range analysis

**Figure 2:**
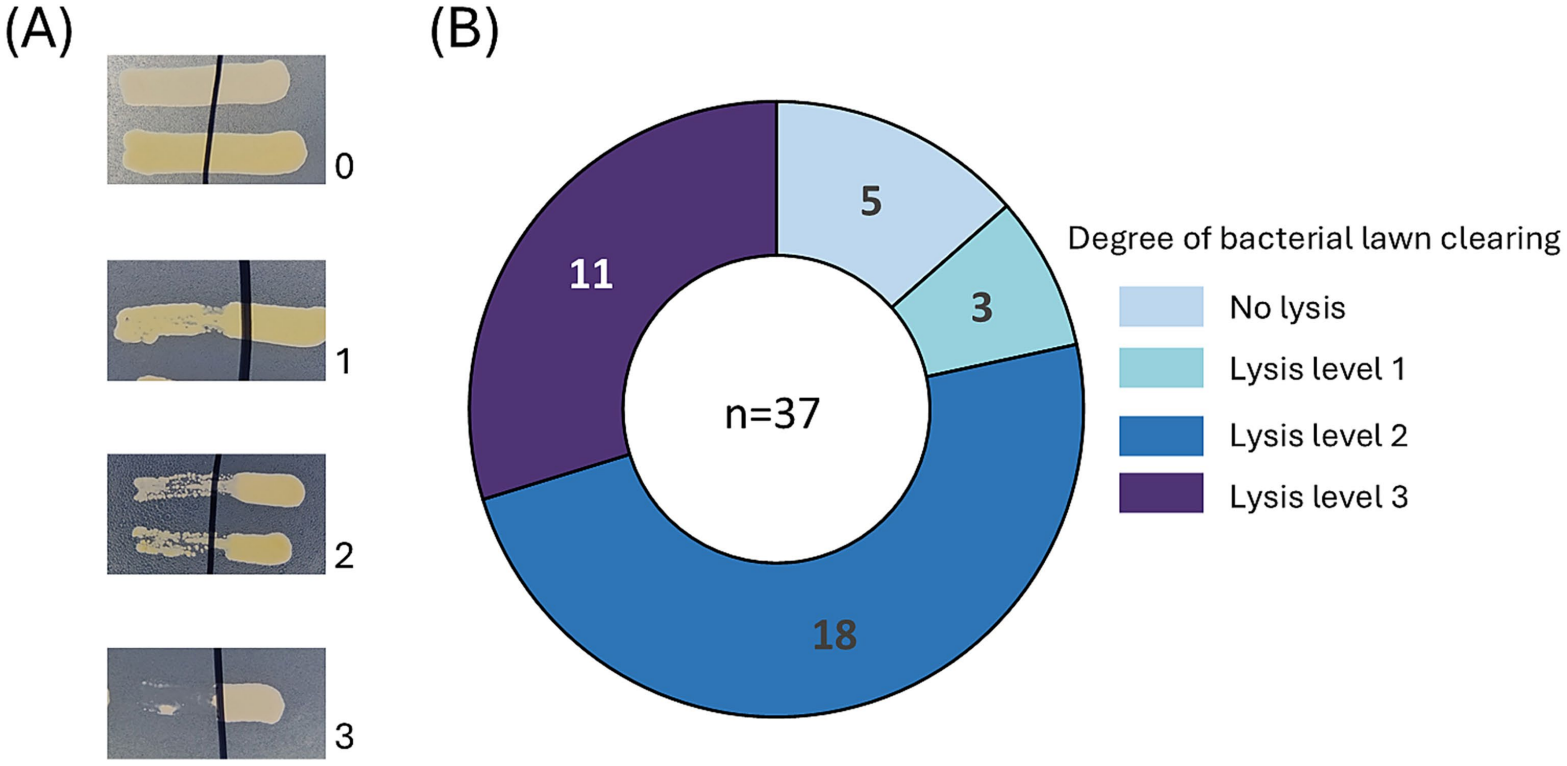
Assessment of phage vB_SauP-INT105 lytic activity by cross-streak assay. **(A)** Representative results of the cross-streak assay, showing different degrees of bacterial lysis classified according to the established scoring system (0–3). **(B)** Distribution of lysis scores obtained in the cross-streak assay, expressed as the number of isolates assigned to each score category.

The host range of phage INT105 was determined by testing its ability to lyse 37 *S. aureus* strains using a cross-streak assay (Figure 2A). The phage was able to degrade the bacterial lawn in 29.73% (11/37) of the isolates tested. An intermediate lytic response, characterized by a partial clearing of the bacterial lawn, was observed in 56.76% (21/37) of the *S. aureus* isolates. In contrast, 13.51% (5/37) of the strains showed no susceptibility to infection (Figure 2B).

### 3.3 Phage adsorption kinetics and one-step growth curve analysis

**Figure 3:**
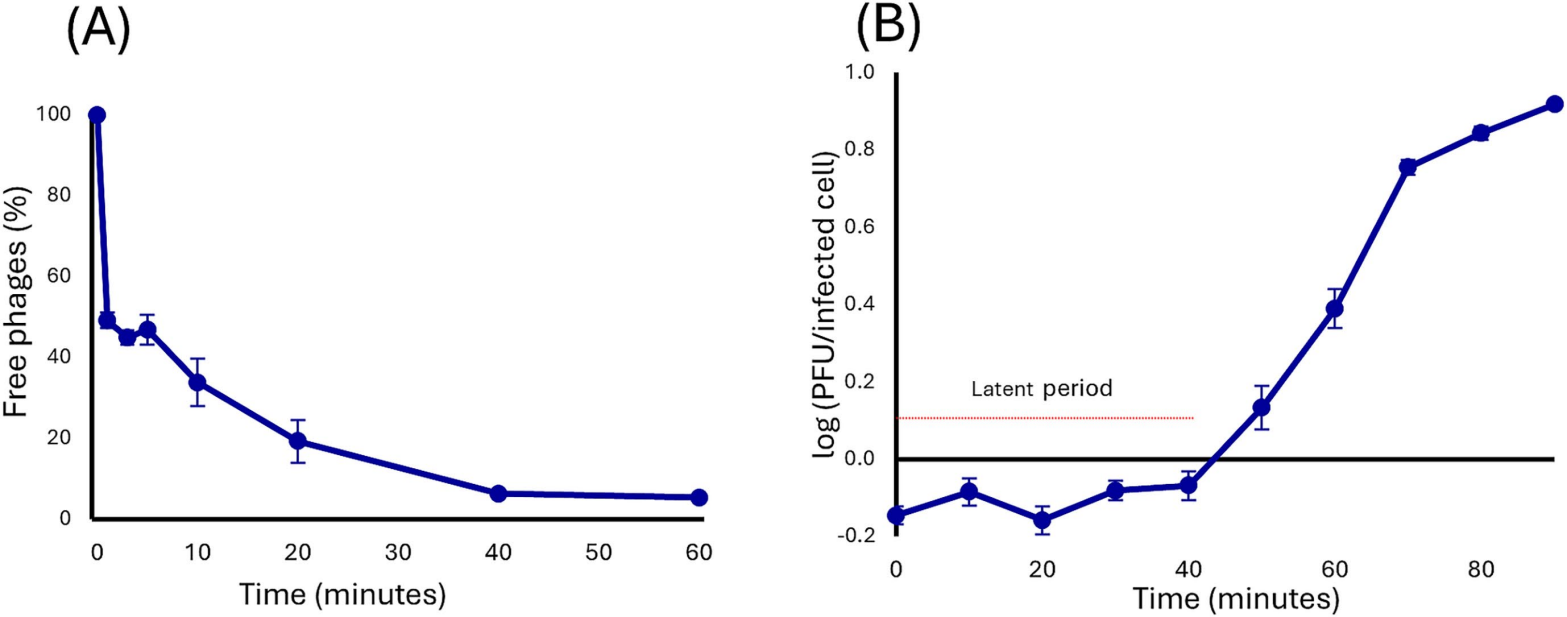
Kinetic characterization of bacteriophage vB_SauP-INT105. **(A)** Adsorption curve showing the percentage of non-adsorbed phages over time. **(B)** One-step growth curve showing phage titers at defined time points. In both panels, the *x*-axis represents the incubation time of phages with host bacterial strain (Sa296). Data are presented as mean values ± standard deviation from three independent experiments.

The adsorption kinetics of phage INT105 on bacterial strain Sa296 was assessed over a 60-min period at an MOI of 0.01 (Figure 3A). A rapid decrease in the percentage of free phages was observed within the first 10 min, with the concentration of unabsorbed phages falling from 100 to 33.85%. This initial phase indicates a high adsorption rate. After 10 min, the adsorption process slowed down but continued to a final free phage concentration of 5.34% at the 60-min mark. The results indicate efficient adsorption of phage INT105 to strain Sa296 under the tested conditions. To further evaluate the complete replication cycle, the one-step growth curve of phage INT105 was determined (Figure 3B). This assay revealed a latency period of approximately 40 min. Following this period, a sharp increase in the phage titer was observed, rising from −0.07 log PFU/infected cell at 40 min to a maximum of 0.92 log PFU/infected cell at 90 min. The estimated burst size was approximately 23 phages per infected cell.

### 3.4 Thermal and pH stability of phage vB_SauP-INT105

**Figure 4:**
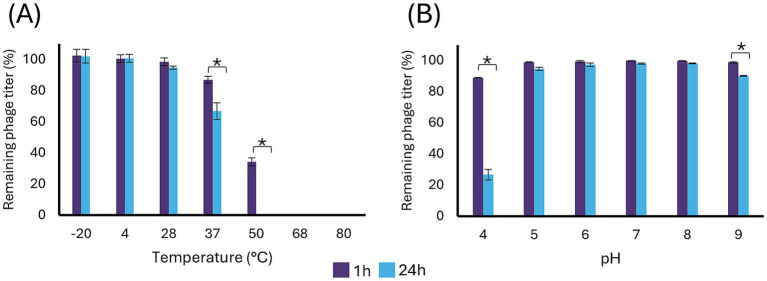
Stability of phage vB_SauP-INT105 under different environmental conditions **(A)** Temperature and **(B)** pH. Phage titers were determined after 1 and 24 h of incubation. Results are expressed as relative phage titers compared to the initial concentration and represent mean values ± standard deviation of three independent experiments. Asterisks above the bars indicate statistically significant differences among groups (*p* < 0.05).

The thermal stability of phage INT105 was evaluated at different temperatures (Figure 4A). The phage maintained significant viability over a range of refrigeration to room temperatures, with optimal stability observed between −20 °C and 28 °C. At 37 °C, the phage showed good short-term stability, retaining 87.0% of its viability after 1 h and 66.7% after 24 h. Stability decreased sharply at temperatures above 50 °C, which was identified as the critical threshold, leading to a complete loss of viability after 24 h. Temperatures of 68 °C and above resulted in immediate inactivation of the phage. To assess long-term storage stability, the phage preparation was maintained at 4 °C and monitored over an extended period (data not shown), demonstrating remarkable stability with only minimal titer reduction: 98.5% retention after 6 months and 97.4% after 20 months. Regarding pH stability, the phage demonstrated optimal stability in a neutral to slightly acidic/alkaline pH range (pH 5 to 8), where it maintained over 98% viability after 24 h. In contrast, viability was significantly affected at both low and high pH. At pH 4, the phage lost 73.4% of its viability within 24 h. In contrast, at pH 9.0, the reduction was lower, with a loss of 9.8% in viability over the same period (Figure 4B).

### 3.5 Genome characterization of phage vB_SauP-INT105

**Figure 5:**
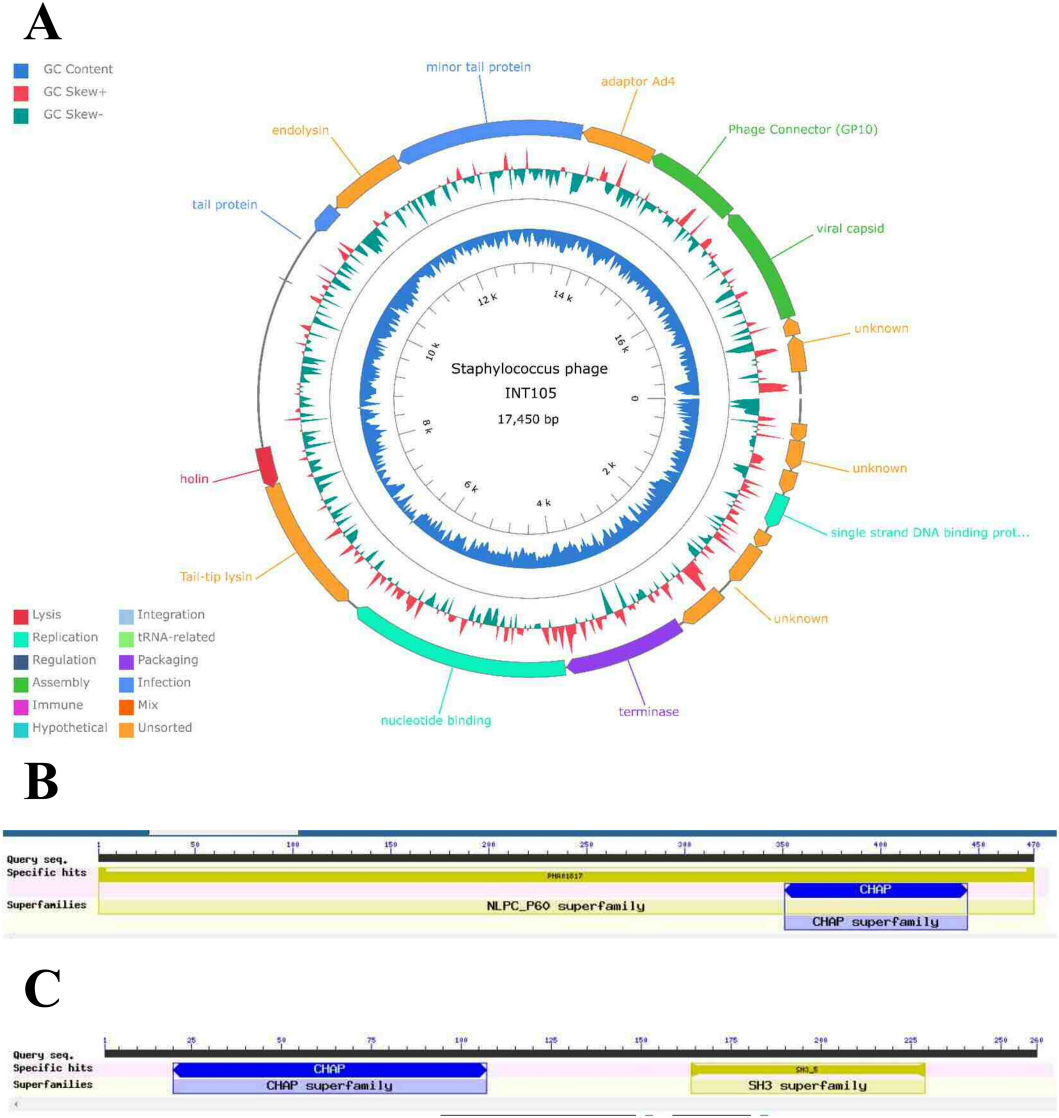
Genomic organization and domain architecture of the phage vB_SauP-INT105 lytic proteins **(A)** Circular genome map showing predicted open reading frames color-coded by functional category (outer ring), GC skew (middle ring), and GC content (inner ring). **(B)** Conserved domain analysis of the vB_SauP-INT105 potential tail-tip lysin, highlighting the presence of a CHAP catalytic domain (NLPC_P60 superfamily) and the absence of an identifiable cell-wall-binding domain (CBD). **(C)** Domain architecture of the putative endolysin, featuring a CHAP catalytic domain and a C-terminal SH3_5-type CBD.

Phage INT105 has a double-stranded DNA genome comprising 17,450 bp (97.33% completeness as determined by checkV), with an estimated GC content of 29.14%, which is slightly lower than that of *S. aureus* (32.17%) (Figure 5A). The genome encodes 20 ORFs (NCBI accession number PZ019817), but no tRNAs, anti-CRISPR, virulence, antibiotic-resistance or lysogeny genes were detected. The predicted ORFs encode structural proteins (capsid, tail, collar), as well as proteins involved in lysis (holin and endolysin), genome replication (DNA polymerase), and DNA packaging (terminase). The lytic protein located next to the holin possesses a cysteine, histidine-dependent amidohydrolases/peptidase (CHAP) lytic domain, but no identifiable cell-wall-binding domain (CBD), thus exhibiting a similar structure to protein GP10 of *S. epidermidis*-infecting phage Andhra (Cater et al., 2017) (Figure 5B). Although its position seems to indicate that it is the endolysin, a recent study on the structure of Andhra revealed that it is actually a tail-tip lysin (Hawkins et al., 2022). In contrast, the lytic protein located next to genes encoding structural proteins has a CHAP domain and an SH3_5 CBD (Figure 5C), a structure that suggests it may be the actual endolysin.

Consistent with the halo morphology observed on strain Sa105, genomic analysis led to the identification of genes coding for proteins with potential exopolysaccharide depolymerase activity. Analysis of the predicted proteome with the DePP tool suggested that the two most likely candidate ORFs to code for this enzyme were ORF12 and ORF15, with respective probabilities of 0.85 and 0.83. Both proteins were annotated as putative major and minor tail proteins, respectively, supporting a potential role in degrading extracellular polysaccharides during the initial stages of infection. Interestingly, the product of ORF15 harbors a knob-like domain, which is typical of pyocins and some phage proteins, including the endosialidase of phage K1F that can degrade the capsule of *E. coli* (Stummeyer et al., 2005).

**Figure 6:**
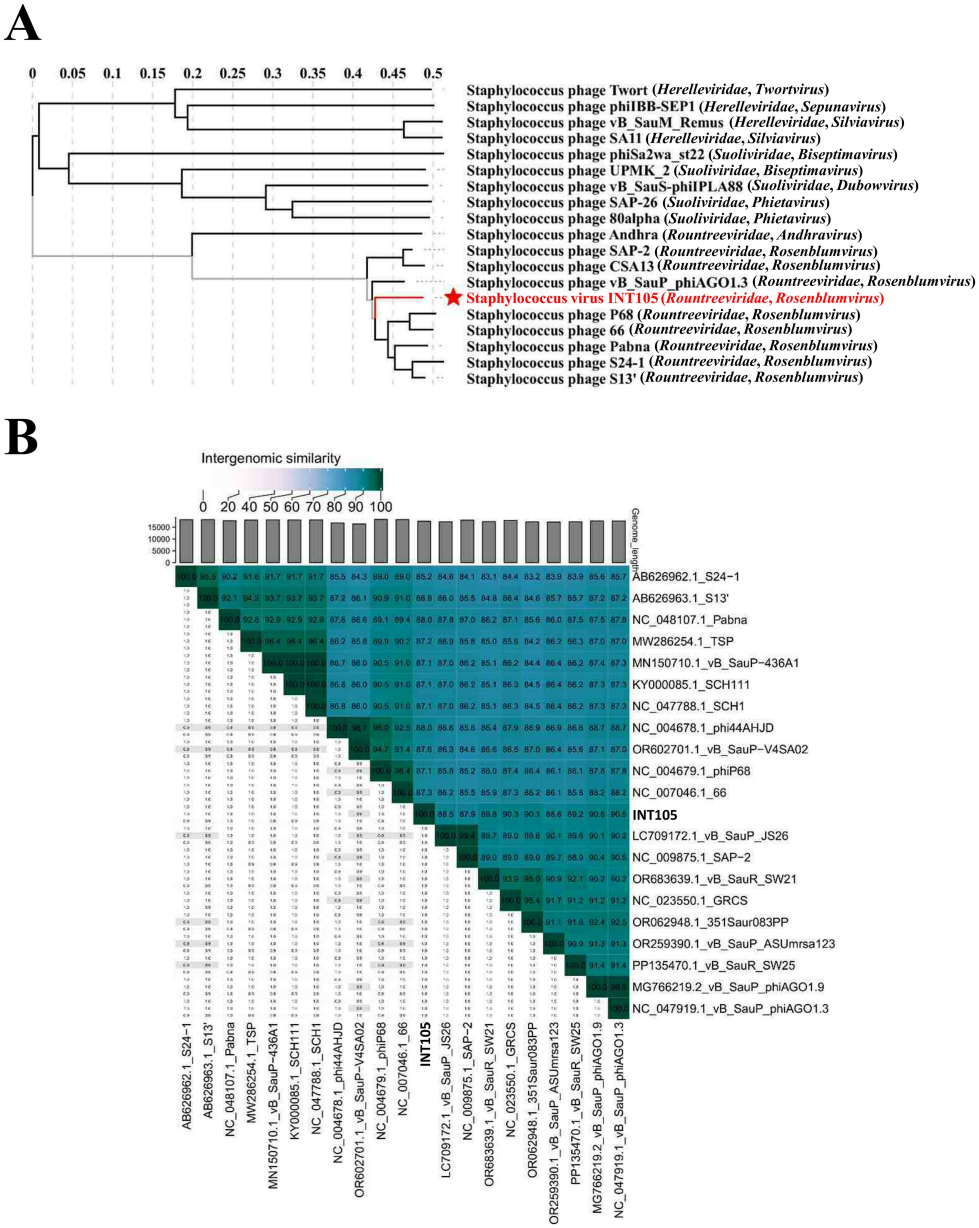
Phylogenomic analysis of vB_SauP-INT105 **(A)** Neighbor-joining tree built from intergenomic distances using VIPTree. The family and genus of each phage is indicated in brackets. **(B)** Pairwise intergenomic similarity matrix comparing vB_SauP-INT105 with related phages.

As expected, INT105 clustered more closely related with other short-tailed phages infecting *Staphylococcus* than with phages of different morphologies (Figure 6A). Its closest relative was phage vB_SauP_phiAGO1.3, with which it shares 90.5% nucleotide identity, placing it within the *Rountreeviridae* family (Figure 6B). Analysis of the taxonomic distance between INT105 and the 20 most similar phages indicated that this phage represents a new species within the *Rosenblumvirus* genus (Figure 6B). Indeed, the intergenomic similarities estimated by VIRIDIC were below the 95% threshold set by the ICTV (Turner et al., 2021).

### 3.6 Antibiofilm potential of phage INT105

**Figure 7:**
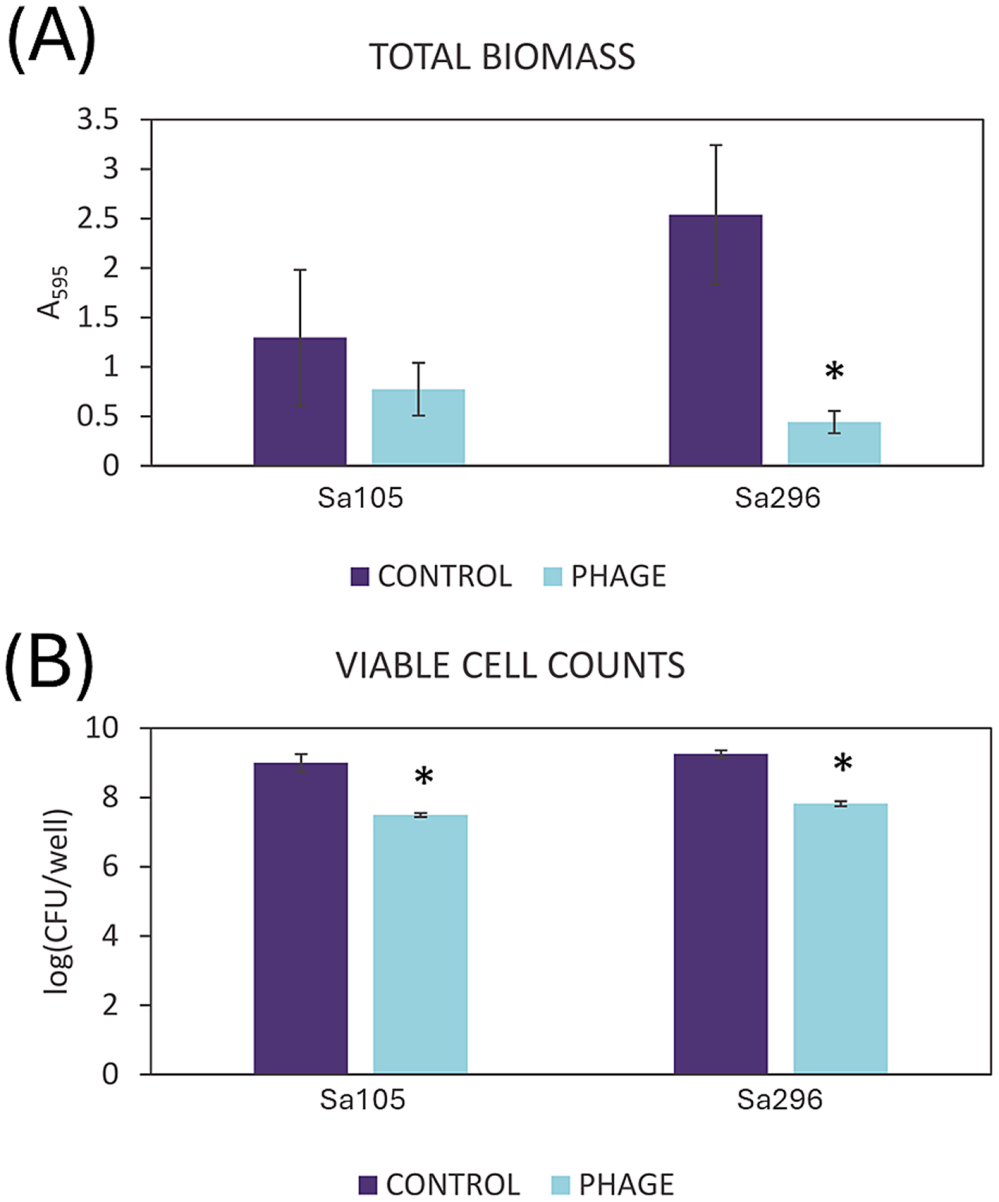
Effect of vB_SauP-INT105 treatment on *S. aureus* biofilms. Twenty-four-hour biofilms formed by strains Sa105 and Sa296 were treated with PBS alone (control) or PBS containing 10^8^ PFU/ml of phage. Following incubation at 37 °C for 24 h, the planktonic phase was removed and all wells were washed and processed equally. **(A)** Biofilm biomass quantified by crystal violet staining and subsequent measurement of absorbance at 595 nm. **(B)** Viable cell counts recovered from biofilms. Data are expressed as mean ± standard deviation of three independent experiments. Asterisks indicate statistically significant differences compared with the untreated control (*p* < 0.05).

Given the relevance of biofilms in both clinical and food-processing environments, we next evaluated the ability of INT105 to act against established *S. aureus* biofilms. The phage was tested on 24-h pre-formed biofilms of strains Sa105 and Sa296, which were selected due to their high susceptibility in host-range assays, although only Sa105 produced the characteristic turbid halo around plaques. In the untreated control condition, strain Sa296 exhibited higher biofilm biomass than Sa105 (Figure 7A). Phage treatment reduced biofilm biomass in both strains compared to the untreated control, although statistical significance was reached only in strain Sa296 (*p* < 0.05). In Sa105, the decrease did not reach statistical significance. In contrast, viable cell counts within the biofilms were significantly reduced following phage treatment in both strains (Figure 7B), achieving an approximate 1.5-log_₁₀_ reduction in CFU/mL relative to the control. These results indicate that INT105 effectively kills biofilm-embedded cells even when overall biomass reduction is limited, supporting its potential as an antibiofilm agent.

## 4 Discussion

In the present study, we isolated and characterized the lytic phage INT105. Its genomic organization and phylogenetic analyses supported its placement within the genus *Rosenblumvirus*, whereas intergenomic similarity values below the ICTV species demarcation threshold, suggest that INT105 represents a putative novel species. Phages with a short-tailed morphology and lytic activity against *S. aureus* are isolated far less frequently than other tailed phage morphotypes (Deghorain and Van Melderen, 2012). Different authors have consistently reported phage collections where siphovirus and myovirus are the dominant morphotypes. Within the *S. aureus* phage system, siphoviruses are commonly associated with a temperate (lysogenic) lifestyle (Ingmer et al., 2019), although the representation of different morphotypes among isolated phages is also influenced by methodological and sampling biases, including host availability and isolation strategies (Kwan et al., 2005; Göller et al., 2021). In this context, the characterization of INT105 contributes to expanding the currently available diversity of lytic *Rosenblumvirus* phages active against *S. aureus*.

Phage INT105 demonstrated broad-spectrum lytic activity against the *S. aureus* panel tested, exhibiting antimicrobial activity (ranging from complete to intermediate clearing) against a high number of isolates (32/37, 86.5%). This observed breadth of activity exceeds the host range values recently reported for other *S. aureus*-infecting *Rosenblumvirus,* which showed lytic activity against approximately 8–75% of tested isolates depending on the evaluated strain collection (Głowacka-Rutkowska et al., 2019; Sharifi et al., 2024; Banar et al., 2025; André et al., 2025). A broad host range is a critical attribute for phage-based applications, as the genetic and phenotypic diversity of *S. aureus* strains can limit the effectiveness of narrow-spectrum phages. The intermediate clearing observed in some strains during the cross-streak assay might be determined by host-related factors that partially restrict phage replication. Such factors may include active and passive defense mechanisms such as restriction-modification and abortive infection systems, or factors affecting the early steps of the lytic cycle, such as receptor availability or capsule interference with adsorption (Egido et al., 2021).

The evaluation of kinetic parameters such as the adsorption rate, the latency period and the burst size is also key to determining the potential of a bacteriophage in biocontrol applications (Costa et al., 2025). In this study, the phage INT105 showed efficient adsorption to *S. aureus* cells, which suggests a strong interaction with bacterial receptors. The latency period of 40 min is within the range previously reported for closely related *S. aureus* phages currently assigned to the genus *Rosenblumvirus* (Kornienko et al., 2020; Sharifi et al., 2024), indicating comparable replication dynamics within this lineage. The estimated burst size of 23 PFU/cell falls at the lower end of the values reported for *S. aureus* phages, which range from approximately 25 PFU/cell to more than 400 PFU/cell (Gutiérrez et al., 2015b; Głowacka-Rutkowska et al., 2019; Cunha et al., 2025; Teng et al., 2022; Banar et al., 2025).

The phage’s stability against various environmental factors is a crucial parameter for assessing its suitability for applications such as biocontrol in the food industry (Pradeep et al., 2022). The results suggest that phage INT105 possesses robust thermal and pH stability, optimizing its potential as a biocontrol agent against *S. aureus* in dairy products. The biological activity of the phage remained stable in the short term at temperatures up to 37 °C, a range essential for milk processing and dairy fermentation. Furthermore, the activity of INT105 was stable after 24 h of exposure to pH values ranging from pH 5 to pH 8. This stability is highly favorable for its efficacy in a dairy context, where the pH naturally drops during fermentation. This is critical for ensuring that the phage remains active during the initial stages of cheese production and for preventing milk spoilage (Wang et al., 2016; Kornienko et al., 2020). The high stability and activity under these conditions strongly support the use of INT105 as a natural anti-staphylococcal agent to enhance food safety and control contamination originating from mastitis-related strains. In that sense, it is also important to highlight that the genome of this phage does not carry any genes related to virulence, lysogeny or antibiotic resistance.

Phage-mediated weakening of the extracellular matrix could facilitate lytic activity by enabling greater diffusion through the biofilm and improved access to binding sites, consistent with evidence that depolymerases and other phage-encoded lytic proteins promote matrix degradation and biofilm penetration (Lu et al., 2023). The plaque morphology of INT105, featuring a clear lytic center and an expanding peripheral halo, is a phenotypic hallmark of phage-encoded depolymerase activity (Latka et al., 2017). This three-zone gradient of decreasing intensity could reflect a complex diffusion dynamic. While the central clearing likely results from active lysis during early lawn growth, the translucent haloes would appear as depolymerases—either virion-associated or soluble—degrade the capsular exopolysaccharides (EPS) of *S. aureus*. The stabilization of the innermost zones at 48 h, contrasted with the continued expansion of the outermost layer, might indicate that enzymatic activity persists after the host bacteria enter a stationary metabolic state. At this stage, the transition of the host into this state could significantly limit further phage-mediated lysis, potentially rendering the infection cycle inefficient (Adams, 1959). Finally, the outermost diffuse zone, which reaches its maximum definition at 144 h but progressively loses boundaries thereafter, might reflect a decline in enzymatic stability coupled with ongoing capsular EPS synthesis by the host, which in *S. aureus* is characteristically sustained and upregulated during post-exponential growth (Sadykov et al., 2010; Rausch et al., 2019). Indeed, this potential depolymerase activity may explain why phage treatment reduced viable biofilm-associated cells in both strains, despite the higher biofilm biomass formed by Sa296 compared to Sa105. Therefore, INT105 may retain antibiofilm activity across *S. aureus* strains exhibiting different biofilm-forming capacities, reinforcing its potential as a biocontrol agent. Furthermore, the ability of INT105 to compromise the biofilm extracellular matrix may support combination therapeutic approaches by enhancing antibiotic or antimicrobial agent penetration and efficacy, a phenomenon that has been demonstrated in prior studies describing phage-antibiotic synergy against biofilms (Topka-Bielecka et al., 2021; Duarte et al., 2024; Kunz Coyne et al., 2024).

In conclusion, the lytic bacteriophage INT105 has demonstrated high efficacy against a broad collection of *S. aureus* strains obtained from dairy goat farms. Its wide host range, notable stability under various environmental conditions, and ability to reduce biofilms position this phage as a promising candidate for biocontrol applications. Genomic analysis confirmed the absence of genes associated with virulence, antibiotic resistance, or lysogeny, supporting its safety profile for potential use in clinical and agri-food settings. Nevertheless, further studies are required to evaluate its scalability and performance *in vivo* and in food-processing systems, as well as its incorporation into phage cocktails to broaden host coverage and minimize the risk of resistance development.

## Data Availability

The datasets presented in this study can be found in online repositories. The genome sequence has been deposited in the NCBI GenBank database and is publicly accessible at: https://www.ncbi.nlm.nih.gov/nuccore/PZ019817.

## Footnotes

1
https://timskvortsov.github.io/WebDePP/
