# Supplementary material for: Phenotypic and genomic characterization of vB_SauP-INT105, an exopolysaccharide depolymerase-encoding lytic short-tailed phage with antibiofilm activity against Staphylococcus aureus
Source: Front Microbiol. 2026 Jun 17;17:1856705. doi: 10.3389/fmicb.2026.1856705 (PMC13319892; doi:10.3389/fmicb.2026.1856705)
Supplement: Supplementary file 1 [file Table_1.DOCX]

**Supplementary Table 1.** *S. aureus* strains used in this study. Colony morphology on Baird–Parker Egg Yolk Tellurite Agar (BP-EYT) was evaluated after 48 h at 37°C. Clear zones and opalescent ring indicate lipolytic and lecithinase activities on egg yolk, respectively (+: present; −: absent; +/−: weak). Coagulase activity was determined by standard assay (+: positive; −: negative).

| **Strain** | **Coagulase test** | **Baird-Parker Agar** | |
| --- | --- | --- | --- |
|  |  | **Clear zone** | **Opalescent zone** |
| Sa103 | + | + | +/− |
| Sa104 | + | + | + |
| Sa105 | + | + | + |
| Sa123 | + | + | + |
| Sa124 | + | + | + |
| Sa136 | + | + | +/− |
| Sa140 | + | + | +/− |
| Sa142 | + | + | +/− |
| Sa143 | + | + | +/− |
| Sa144 | + | + | + |
| Sa145 | + | + | + |
| Sa150 | + | + | +/− |
| Sa151 | + | + | +/− |
| Sa155 | + | + | +/− |
| Sa156 | + | + | +/− |
| Sa157 | + | + | +/− |
| Sa178 | + | + | + |
| Sa179 | + | + | + |
| Sa181 | + | + | +/− |
| Sa183 | + | + | + |
| Sa184 | + | + | + |
| Sa185 | + | + | - |
| Sa186 | + | + | + |
| Sa187 | + | + | + |
| Sa188 | + | + | + |
| Sa189 | + | + | +/− |
| Sa190 | + | + | + |
| Sa191 | + | + | + |
| Sa193 | + | + | +/− |
| Sa194 | + | + | + |
| Sa196 | + | + | + |
| Sa199 | + | + | + |
| Sa200 | + | + | + |
| Sa201 | + | + | + |
| Sa292 | + | + | + |
| Sa296 | + | + | + |
| Sa301 | + | + | + |
